# Choreographing life-experiences of balance control in people with Parkinson’s disease

**DOI:** 10.1186/s12883-020-01632-4

**Published:** 2020-02-10

**Authors:** Sofie LaGrone, Conran Joseph, Hanna Johansson, Birgit Enberg, Erika Franzén

**Affiliations:** 1 Department of Community Medicine and Rehabilitation, Umeå University, Umeå, Sweden; 2 Department of Health and Rehabilitation Sciences, Division of Physiotherapy, Stellenbosch University, Cape Town, South Africa; 3 Department of Neurobiology, Care Sciences and Society, Division of Physiotherapy, Karolinska Institutet, Stockholm, Sweden; 4 Karolinska Institutet and Function Area Occupational Therapy and Physiotherapy, Function of Allied Health Professionals, Karolinska University Hospital, Stockholm, Sweden; 5 Stockholms Sjukhem R&D unit, Stockholm, Sweden

**Keywords:** Parkinson’s disease, Balance control, Activities in daily life

## Abstract

**Background:**

Parkinson’s disease (PD) is a devastating neurodegenerative disorder. Reduced balance is one of the cardinal symptoms of PD, predisposing people living with PD to experience difficulties with the execution of tasks and activities, as well as hindering their involvement in meaningful life areas. The overarching aim of this study was to explore how deficits in balance control manifest in everyday life and how it is managed by people with PD (PwPD).

**Methods:**

Qualitative description was used as methodology, and in-depth interviews were conducted with 18 participants, between the ages of 46 to 83 years, with mild to severe PD. Interview transcripts were analyzed using qualitative content analysis, following an inductive approach.

**Results:**

One theme emerged from the analysis: *Increased planning—choreographing life*. Within this overarching theme, two categories were identified, namely *Limitations in mobility* and *New restricted functioning in everyday life,* each with 3–4 sub-categories. The categories described how PwPD handled decreased balance control in their everyday life by using motor and cognitive strategies as a consequence of not trusting their body’s capacity to control balance. Activities in everyday life, as well as the ability to partake in leisure and social activities were profoundly affected.

**Conclusion:**

People with mild to severe PD used strategies to handle decreased balance and they choreographed their lives around their individual current state of mobility and balance. The knowledge gained from this study can be used to develop targeted interventions addressing the nuances of balance deficits in everyday life.

## Introduction

Parkinson’s disease (PD) is a neurodegenerative disorder for which there is no cure. Despite the variety of pharmacological and non-pharmacological treatments available [1, 2], PD usually results in severe disability the longer an individual lives with the disease. Periods of decreased motor function (off-periods) are common as the disease progresses due to altered response to pharmacological treatments [1].

Postural instability, a hallmark feature of PD, predisposes people living with the disorder to experience difficulties with the execution of tasks and activities [3]. Gait and balance difficulties are complex in PD and are evident early in the disease and progresses over time [4–6]. Balance impairments such as anticipatory postural adjustments, sensory integration and dynamic gait are affected early in the disease [7, 8], often resulting in a higher occurrence of falls in people with PD (PwPD) than healthy controls even before diagnosis [9]. Reduced balance can also lead to a decreased involvement in life situations, i.e. *participation* as defined by the World Health Organisation [3]. Thus, the use of exercise to increase and maintain balance control has become an important target in rehabilitation [10, 11].

An individual’s balance control is influenced by many bodily systems [12]. Several of these systems, such as sensory integration, reactive responses, anticipatory postural adjustments, functional limits of stability, motor agility and dual-tasking, are affected in PwPD, which result in reduced balance control [4, 5, 13–15]. To perform dual-tasks, the ability to divide attention and perform collective motor and cognitive tasks is needed [16, 17]. The latest recommendations from expert guidelines suggest the maintanance of functional abilities through supervised exercise training which challenge the limits and capacity of PwPD [18, 19]. High level evidence, derived from rigorous randomised controlled trials, exists, which indicate the beneficial effects of training on balance performance and gait [20]. However, studies have also found that objective improvements in balance and gait did not always align with perceived improvements, especially within the context of everyday living [19]. Furthermore, participants felt that their balance and functional abilities were non-modifiable and could only decline over time. These findings necessitate the research community to investigate beliefs and attitudes with respect to the complexity of balance control in Parkinson’s disease using the insiders’ perspective.

To date, very few studies have explored how PwPD perceive balance control and the meaning they ascribe to it. There is a definitive need to understand how PwPD conceptualise balance control and how it impacts their everyday life. Moreover, there is a need to identify strategies PwPD use to counteract balance deficits. Findings from such studies may, in turn, address misconceptions surrounding the modifiability of balance control and lay the foundation for developing targeted interventions that address the nuances of balance deficits in everyday life. The ***aim*** of this study was to explore how deficits in balance control manifest in everyday life and how it is managed by PwPD.

## Method and design

Qualitative description was used as the research method due to its ontological origin within the doctrines of subjectivism and nominalism [21, 22]. Qualitative description is a useful method for understanding the lived experiences of people around a particular phenomenon where multiple unique experiences exist [22]. It provides a basis for developing a conceptual understanding of the meaning and experiences attached to life situations [22]. The study was approved by the Regional Ethical Council of Stockholm (registration number of original application 2016/201–31 and supplement application 2016/1973–32). Prior to the start of the study, participants were given verbal and written information about the study before written consent was obtained. Participants only enrolled in the study once written informed consent was provided.

### Participants and setting

Participants were recruited from the area of Stockholm County in Sweden. The aim was to include a wide range of PwPD, who where different in terms of their age, sex, living status, years since diagnosis and disease stage. The inclusion criteria were: 1) a diagnosis of idiopathic PD [2], 2) ability to communicate in swedish and 3) self-perceived reduced balance. Exclusion criteria was scores of ≤21 on the Montreal Cognitive Assessment (MoCa) [23]. Nineteen participants were recruited to the study, where one was excluded due to scoring low on the MoCA test. The median age and range of this sample was 70 [46–83]. All of the participants perceived reduced balance when initially questioned, and it was clinically assessed using the Mini-Balance Evaluation Systems Test (Mini-BESTest) [24, 25]. Further, the Activities and Balance Confidence (ABC)-scale [26, 27], the Walk-12G scale [28], the motor examination part (III) of the Movement Disorders Society- Unified Parkinson Disease Rating Scale (MDS-UPDRS) [29, 30] and classification by Hoehn & Yahr (H&Y) score between 1 and 4 (from minimal to severe disability) [31] were used to collect background information and is presented in Table 1.

**Table 1:** Background data of participants (*N* = 18)

ID No	Gender	Years since diagnosis	Education ^a^	H&Y ^b^	MDS-UPDRS ^c^	Mini BesTest ^d^	ABC scale (%) ^e^	Walk 12 ^f^
1	Female	21	3	3	40	24	60.0	15
2	Male	10	3	3	46	20	86.9	0
3	Male	4	3	2	40	16	83.1	16
4	Male	5	2	2	31	11	73.1	25
5	Female	10	3	2	14	–	–	0
6	Female	4	3	2	19	24	76.3	8
7	Female	2	3	3	28	6	16.3	17
8	Female	7	3	1	16	20	70.6	1
9	Male	2	1	2	32	17	51.9	15
10	Male	14	2	3	33	22	64.3	19
11	Male	21	3	2	32	19	76.3	14
12	Male	9	3	3	23	19	75.6	23
13	Female	7	3	2	18	24	72.5	25
14	Female	3	2	2	22	19	26.3	28
15	Female	11	3	3	29	16	72.5	24
16	Male	10	3	4	62	4	16.9	38
17	Male	13	1	2	24	28	83.1	6
18	Female	5	3	2	20	25	88.8	2

^a^Level of education 1 = Junior high school degree, 2 = High school degree, 3 = University degree

^b^Hoehn & Yahr, a five-point disease rating scale, 1 = minimal or no disability and 5 = confinement to bed/wheelchair

^c^Movement Disorder Society -Unified Parkinson Disease Rating Scale, part (III), motor disability maximum score 56

^d^A 14-item clinical test covering 4 components of balance control maximum score 28

^e^Activities and balance confidence scale, a 16 item self-report questionnaire total score 100%

^f^The walk 12 scale, a questionnaire about walking difficulties in everyday life, total score 42

The participants were tested in an ON medication state. Eight of the participants used some type of walking aid for some part or the entire day. Twelve of them had experienced one or more falls in the last year. Four people lived alone, of which all were women. Five people lived in houses while the rest resided in apartments. Only three persons had assisted living services and six used household services. All of the participants used medication for PD; 17 participants used Levodopa of which one had a duodopapump, six participants used dopamine agonists, four participants used MAO-B, three participants used COMT inhibitors and one participant undergone deep brain stimulation.

### Data collection

The method used for data collection was in-depth interviews. The interviews were performed during November 2016 to February 2017 by a researcher and physical therapist (HJ). Interviews were conducted face-to-face in a location of the participant’s choice (mostly home-environment), and lasted between 20 to 70 min. A semi-structured interview guide with open-ended questions was used [32]. The first question in the interview guide was “If you were to describe to somebody that doesn’t know what it means to have PD, how a common day in your life is, what would you tell that person?” The other question areas were the concept of balance, activity and participation perspectives, falls and/or fear of falling and dual-task situations. The last question was “Is there something you think that we haven’t talked about regarding balance and falling, or is there something that you would like to ask about?” For further information, see Additional file 1. The interviews were recorded and then transcribed verbatim. Interviews were performed before testing and collection of descriptive data, ensuring that inquiries were done with minimum disruption of the researched phenomenon and without bias of the participant’s original ideas and thoughts regarding the researched area.

### Data analysis

Qualitative content analysis was performed according to Graneheim & Lundman, with an inductive approach, since previous research in this area is limited [33–35]. The material, i.e. individual protocols, was read several times to ensure familiarization of the data [34]. Meaning units were selected and then condensated [33]. After that, abstraction and interpretation on a higher level was attempted and applied [35]. The meaning units then received a code, i.e. a label. The codes were then sorted into different sub-categories and then categories. Lastly, a latent theme that connected the underlying categories together was identified. Both latent –the underlying meaning, and manifest content –the visible were included in the analysis [33]. Examples of the analysis process are shown in Table 2. The collected descriptive data and other contextual information were also taken into consideration so that a holistic analysis could be performed [36, 37].

**Table 2:** Example of the analysis process

Meaning unit	Condensed meaning unit	Code	Sub-category	Category	Theme
Yes it, from day to day. Yes, it can change, in one half-hour I may not be able to walk and then it changes back then again and then I can be as movable as ever before.	From day to day. One half-hour not able to walk and then back again and then be as movable as ever before.	Mobility changes from one moment to another.	Planning for fluctuating symptoms	Limitations in mobility	Increased planning—choreographing life
Yes then you have to be a little, either grab a hold in the arm of my wife or take a hold of the cane or move myself sideways so that I pass by it.	Either grab a hold in the arm of my wife or the cane or move myself sideways pass by it.	Can’t walk over dangerous surfaces, avoids them or needs extra support.	Increased attention to navigate uneven terrain		
But it doesn’t affect that much. It affects me because I am less I am more limited, I cannot do, not be free as I was before.	Doesn’t affect that much. Affects me more limited, cannot be free as before.	Can no longer be spontaneous.	Restricted personal & social freedom	New restricted functioning in everyday life	
Yes, what should I say … it’s okay. It is … yes, so here in the apartment then you can do almost everything except climbing up on any ladders and such, that I avoid because then you can fall and hurt yourself.	It’s okay. In the apartment you can do almost everything except climbing on any ladders and such, I avoid because you can fall and hurt yourself.	Can do all domestic chores in the apartment except walking up on ladders	Strategies to perform everyday tasks with less trepidation		

Several steps were taken to increase the trustworthiness of the study [38]. To check for conformability of data, continuous re-reading was carried-out to confirm that meaning units, categories and themes were truthfully represented from the data [33]. The data analysis was conducted by the first author (SL), a physical therapist with clinical experience of working with PD as well as other neurological diseases and geriatrics. To enhance credibility and representativeness, peer-debriefing sessions were held with co-authors (HJ, BE, and EF) until agreement was reached [38].

## Results

The overarching theme derived from the data was: *Increased planning—choreographing life*. Within this overarching theme there were two categories, namely *Limitations in mobility* and *New restricted functioning in everyday life,* each with either 3 or 4 sub-categories (see Table 3). The first category examined how performance of everyday movements were affected and how certain strategies were used to stay in control with respect to participants safety and resources. The other category explored how diminishing balance control altered participation in activities in their daily and social lives. Moreover, this category expands on how participants applied strategies to perform everyday tasks to maintain independence and functioning. The participants tended to rigorously plan their life around their current state of mobility and balance. Overall, there was also an increased need for awareness in order to remain in balance and a need to choreograph each of their movements and activities.

**Table 3:** Description of theme, categories and sub-categories

Sub-categories	Categories	Theme
-Planning for fluctuating symptoms	Limitations in mobility	Increased planning—choreographing life
- Public space and increase of walking limitations		
- Conscious movement strategies		
-Increased attention to navigate uneven terrain		
-Increased tendency to avoid leisure activities	New restricted functioning in everyday life	
-Restricted personal & social freedom		
-Strategies to perform everyday tasks with less trepidation		

### Limitations in mobility

This category explores the increased attention and awareness needed to walk and move safely at home, in public space and in nature. The participants used strategies like planning for fluctuating mobility and increased their awareness to perform everyday movements with a focus on maintaining balance control. It further specifies the increased challenge that different exterior surfaces pose to PwPD.

#### Planning for fluctuating symptoms

As a result of fluctuating mobility participants had a feeling of uncertainty towards their body’s ability. At any moment, periods of reduced mobility could appear that would affect their postural stability. This could mean that participants suddenly need support to stay in balance or that walking would be more unsteady and slower on certain days. However, participants could also predict when fluctuating mobility and balance would occur, and experienced that circumstances like being tired, stressed or cold would exacerbate these symptoms.

*“So sleep is extra important for me, if I don’t sleep enough also my balance is affected … ” Participant 12*

Balance control was experienced to be best after having exercised and when they were well medicated, and was worst during off periods, i.e. periods of bradykinesia. Consequently, the ability to control movements was then reduced, affecting how different functional movements, like standing up to stair climbing were performed. Dissimilarly, periods of dyskinesia could be perceived as easier to control than bradykinesia. Not knowing when periods of reduced mobility would come affected the ability to participate in everyday activities, as they needed to plan for periods of reduced mobility.

*“I haven’t used public transport in ages. Yes due to balance and everything. When I sit down, I can sit down in the car and be so damn alert … and then when I walk out of there then I can hardly get out because I am so stiff. That’s this disease.” Participant 14*

#### Public space and increase of walking limitations

Several scenarios were described when participants had fallen or been close to falling in situations out in the public. Walking inside and especially in stores would trigger walking disabilities, and participants would therefore become fearful of navigating interior and cluttered spaces. Some used a shopping cart or extra medication to be able to perform these tasks.

*“Yes, people are in the way. I notice it in stores when I walk. It’s like coming inside after having been outside and walked with long nice steps outside and then you come in and then it’s short steps and that is how it is in stores also, even though the space is bigger.” Participant 11*

Other situations that would cause problems was walking in crowded areas, like when using public transportation. To walk through a crowd and handle the unpredictability of other people would offset the rhythm of their gait. Walking through narrow spaces like turnstiles on the subway, the doors of a train or getting on the escalator would cause problems with initiation of gait, freezing symptoms and at times cause falls.

*“Last time when I was walking on the escalator last Saturday and was going up, then I had a gait-stop and I wanted so much to get to the escalator but I wouldn’t quite reach it but I thought if I can grab this railing with my hand then it will come, then it pulls me away. But the legs didn’t follow … ” Participant 13*

#### Conscious movement strategies

This sub-category describes how participants had a heightened awareness towards their performance of everyday movements like raising from a chair, standing and turning around. A similarity among participants was having problems with turning in place, also for those that did not experience major difficulties with their balance per se.

*“I can not move, neither turn, just take the foot and turn and put it there, without any drop-downs on support points. It goes very slow when I turn … ” Participant 9*

Also, less complex but common movements in everyday life were considered unsafe or worrisome. Participants were aware of when and how they needed extra support. The need for support when walking varied from using walking aids, such as a walker to walk safely or to holding on to objects in their home environment, to not needing any support at all. However, the walker was also described as an extra “thing” to handle, requiring attention, therefore, not always making walking easier. Something participants had reflected on was the need to concentrate on what they were doing, to think about how they were moving—a strategy used all the time or only in medication off-periods.

*“When you are out and walking otherwise, you just walk. You don’t think, you don’t go on and shout orders to your feet when you walk they just go where you want them to. I kind of need to concentrate on what I am about to do.” Participant 9*

*“Well, when I am about to sit down, a thought can hit me and then I stay standing. I let go of the thought, and focus on the task.” Participant 16*

#### Increased attention and focus to navigate uneven terrain

Participants were afraid of walking on ice and snow during winter and they used strategies, like using ice-cleats to reduce their fear and to try and remain active. Use of walking aids like canes, Nordic walking sticks or having company, were common strategies to feel safer when walking throughout the year. Another strategy was being more mindful by paying close attention to the movements and condition of the surface they were navigating, to avoid slick patches and falls. Participants also avoided going outside completely due to fear of falling, affecting the ability to participate in activities especially during winter-time.

*And I make sure that I use ice-cleats or sturdy shoes so that ... I plan how I will walk and then I take a stroll here in the area and look at curtains and such.” Participant 15*

It was also increasingly harder to walk on uneven terrain like in the woods and on hilly footpaths, since they did not trust their ability. There was a constant need to look ahead on walking paths to have the best grip and to avoid obstacles.

*“If I walk on some trail and meet somebody and so on. Then I can let people pass instead of, so that I don’t have to walk to the side because then I can be afraid of maybe falling or so … ” Participant 6*

### New restricted functioning in everyday life

This category explore how the disease increasingly restricted the participants personal freedoms. Further, this category highlights how participants employed strategies to perform increasingly precarious everyday tasks and how they changed their participation in activities.

#### Increased tendency to avoid leisure activities

This subcategory relates to how PwPD increasingly avoided leisure activities that involved challenging their balance as the disease progressed. One complex activity that participants had ceased with was downhill skiing, due to its complexity and the high risk of falling, but also due to having a disease and natural aging.

*“I feel unsteady and I don’t ski which I did before and that is also connected a little bit with balance but also to, yes, with the whole situation I guess you can say.” Participant 5*

Participants would still try to go cross-country skiing, but mainly on flat surfaces where they could control their own speed and the risk of falling was minimal, while others stopped with this altogether. Participants continued with activities like playing tennis or golf where they adjusted their way of playing to match their physical capacity. Other activities where participants noticed that the balance had changed were when dancing or riding a bicycle.

*“I have a bike that I don’t dare to use any longer. It’s like with an airplane, the critical moments is the take off and landing … I don’t trust my ability.” Participant 8*

Moreover, reduced ability to walk on uneven surfaces especially when simultaneously performing other tasks made it precarious to do work in the garden.

#### Restricted personal & social freedom

This sub-category coincides with how reduced balance have an impact on participants’ emotional and social life. Restricted gait and fluctuating mobility made it harder to be able to plan and attend to different events, as well as keeping social appointments. This could result in participants being confined to their home or them requiring outside company in order to participate. The participants could no longer be spontaneous and this created a feeling of restricted personal freedom. Further, not being able to do the same activities as before, like visiting the town and walk around due to decreased balance capacity, had an effect on their mood.

*“I was free to go to the store and try on clothes or shoes. Go and look at something, an exhibition or concert. Meet friends and go to the movies or organize something and go together but now it is … ” Participant 1*

Gradually the personal freedom and social life was increasingly restricted. As the disease progressed, balance control was no longer taken for granted. On the other hand, by planning beforehand and having support from others, participants could remain active.

*“Now, there will be a Christmas show, then grandpa has always attended so I want to do the same for all of my children. If I can only sit down... then I have one of those chairs … a beach chair that I sit on. Then I can sit for a long time. If I want to it is possible to solve.” Participant 3*

#### Strategies to perform everyday tasks with less trepidation

This subcategory relates to the increased amount of planning and the need for strategies to perform everyday and personal tasks with less fear and anxiety. Depending on the disability level and the prevalence of fluctuating mobility, tasks and activities in the participants’ home, even those concerning their personal hygiene, became increasingly precarious. They were required to be more aware and use extra attention when getting dressed. Another situation performed with apprehension and increased focus due to its complexity, was when participants stood up and attended to personal care after using the toilet.

*“The transition between sitting to doing things, make you afraid. To handle the clothes. To clean up and so on, busy with both your hands. I question my balance. Is it enough? Will I manage?” Participant 10*

Another strategy dealing with fluctuating mobility, was to postpone certain tasks or plan to perform them mainly during on-periods. Off-periods slowed down both movements and cognitive capacity, which affected the ability to perform tasks.

*“Then I can do everything, I have a list of things to do.” Participant 13*

*“If I sleep a little long, then … If I get up at five o’clock, then there is speed in me. Then I go up and make coffee and fix everything … But if I let it get to around seven o’clock, then I can get standing in some situation so that I tense up. The muscles all turned on I hold on to the door, or some table there, something and I don’t dare to let go because then I will take off.” Participant 11*

Depending on how frequent and predictable the off-periods were, other strategies like using aids or getting assistance from someone were needed to assure that a task could be performed. Another increasingly more common strategy was to start “doing one thing at a time”. Participants reflected on the fact that they tended to only do one thing at a time and that it had been a gradual shift.

*“I can feel that when it becomes too much things that should happen then I can say ‘stop now, now let’s take it one thing at a time’. I can say that more often then what I did before.” Participant 6*

## Discussion

Findings from this study suggest that people with mild to severe PD cultivate strategies to function in everyday situations as a consequence of distrusting their body’s capacity. One of the study’s main findings is the need for planning as a consequence of the unpredictability of motor symptoms and balance control. To continue to perform everyday tasks, participants scheduled activities during on-periods of medication. Motoric demanding leisure activities were sometimes avoided due to the unpredictability of balance dysfunction. Participants attributed other motor symptoms like bradykinesia as well as psychological causes to affect their balance control. Moreover, this resulted in a restriction of personal freedom and participation in activities.

Studies exploring the subjective experience of PD symptoms are warranted in order for therapist to better understand patient perspectives. A previous study found that impaired balance in PwPD was perceived as a loss of control in everyday life [39]. In the current study, we unfold the concept of balance further by describing how PwPD experience and manage the consequences of impaired balance. One consequence was the use of planning as a strategy, which was confirmed in other research [40, 41]. Additionally, the results from this study suggest that participants use of planning as a strategy was ever-present. There is also some evidence to suggest that PwPD have an increased personality of harm-avoidance behaviour [42, 43], which may contribute to the use of planning as a strategy and breadth of life involvements. Another strategy that was common among participants in this study was taking additional medicine in times when there seemed to be higher demands on balance and mobility, like when walking in a grocery store. This strategy was also found in a study researching the significance of walking in PwPD [44]. Increased attention and focus on the act of walking was a different, yet effective strategy to stay in balance. In line with this, another study described how persons would “drive” their walking pattern by increasing their attention [45]. Moreover, findings from this study suggest that increased attention were used to “drive” movements in everyday life situations as well.

Both motor demanding leisure activities and activities of a social character were restricted or avoided due to reduced balance. The experience of restricted personal and social freedom is confirmed elsewhere [40, 41, 44]. Previous research shows that decreased mobility, balance, occurrence of freezing of gait (FoG), falls and fear of falls affected daily life and had an impact on participation in different activities [46]. In fact, studies have shown that two risk factors of falling in PwPD, and even healthy elderly, involved reduced balance confidence and concerns about falling [47–49]. Almost all participants reported an increased level of cautiousness, especially during medication off-periods, which can be interpreted as having reduced balance confidence.

Another important finding of this study suggests that an additional dual-task seemed to be very attention demanding. Problems with reduced balance and increase of FoG in dual-task situations (like when walking in crowded spaces and doing grocery shopping) were often described. This could be explained by the fact that regulation of gait variability and rhythmicity is attention demanding for PwPD, and is more affected when a dual task is performed [50]. Moreover, reduced balance when attention is diverted have been reported in people in early stages of PD [6], implying the need to adress balance control as a matter of urgency in the newly-diagnosed. Further, dual-tasking can increase the risk of falling and affect the performance of turning in place and trigger FoG [51–54], which was reported among participants. Dual tasking is considered to be important during activities in everyday life and can be impaired in PD due to motor-symptoms and impaired cognition [16]. Participants in this study reported difficulties when getting dressed, when using the toilet and when cooking. They would increase their attention and prioritize the movement or walking above additional tasks. Further, they were aware of their own capacity with regard to environmental demands and how mobility aids, for example, decreased their functional performance at times.

One strength of the study was the number of participants that were included. Theoretically, this ensures a level of saturation and a deeper analysis of the complex phenomenon –balance control [55, 56]. The interviewer had never met any of the participants before and was presented as a researcher and not as a physical therapist, all aiding in reducing response bias. One limitation was the fact that only one interview, at one point in time, was conducted, leaving a gap concerning the development of balance issues and how it affected functioning over time in PwPD. People in early stages of PD were included in this study since according to the latest research, balance deficits are common even in early stages of PD [4–6], which was also the case in our study. According to our testing 11 of 18 participants had a H&Y score of 1–2, and 10 of these had reduced balance according to one or more domains on the Mini-BESTest. Moreover, six of the participants in early stages H&Y score 1–2, had a score < 21 on the Mini-BESTest which is a predictor for falls [57]. The participants were tested in an on-state of medication, which could explain why one participant, with a maximum Mini-BESTest score of 28, still perceived reduced balance. It would therefore have been interesting to have tested the participants also in the off medication stage. Another limitation was that we did not investigate if the participants had psychological symptoms like depression, that may have negatively affected how they coped with reduced balance. Our earlier research have shown a relation between fear of falling and depressive symptoms [58]. To increase credibility, the analyzed material was peer-debriefed by other authors (HJ, EF and BE) at several occasions [38]. There was an equal amount of men and women included in the study. However, all men lived with somebody while only four of the women did. This may have had an impact on domestic life, where tasks could be divided or shared to a greater extent. It is also important to note that other contextual parameters, like age and disease severity, may have had a larger impact than gender. There may be some limitations concerning the transferability of findings to rural settings due to the fact that all participants lived in the city. However, the aim of this study was not to generalize the results, but rather to develop a conceptual understanding of balance control and how it impacts the lived experience of PwPD.

In conclusion, people with mild to severe PD experience reduced balance and this affects their ability to participate in activities of everyday life and perform dual-tasks. Participants were cautious, avoided difficult tasks or performed them with increased attention. This resulted in participants choreographing their life around current states of mobility and balance. The awareness of having reduced balance was a mind-set with which the participants undertook every activity or task. To be able to handle and perform everyday tasks, movements and activities, participants used motor and cognitive strategies. The knowledge gained from this study can be used to inform PwPD on the variable nature of balance control and performance. Further, it can be helpful in developing targeted interventions that address the nuances of balance deficits in everyday life. Future research is needed on how to implement these strategies, who to target specifically, and when to target them. Furthermore, a need exist to explore how men and women experience reduced balance and how it affects them in daily life. Further work is also required to study the influences of balance control in the context of everyday living and not in the laboratory.

## Supplementary information

**Additional file 1.** Interview guide

## Data Availability

The datasets used and/or analysed during the current study are available from the corresponding author on reasonable request.

## Abbreviations

ABC-scale: Activities and Balance Confidence scale
FoG: Freezing of gait
H&Y: Hoehn & Yahr
MDS-UPDRS: The motor examination part (III) of the Movement Disorders Society- Unified Parkinson Disease Rating Scale
Mini-BESTest: Mini-Balance Evaluation Systems Test
MoCa: Montreal Cognitive Assessment
PD: Parkinsons disease
PwPD: People with PD
