# Supplementary material for: Choreographing life-experiences of balance control in people with Parkinson’s disease
Source: BMC Neurol. 2020 Feb 10;20:50. doi: 10.1186/s12883-020-01632-4 (PMC7008524; doi:10.1186/s12883-020-01632-4)
Supplement: Supplementary file 1 — Additional file 1. Interview guide [file 12883_2020_1632_MOESM1_ESM.docx]

**Appendix 1.**

**Sample of interview questions**

- If you were to describe to somebody that doesn’t know what it means to have PD, how a normal day is like for you, what would you talk about?
- Can you give examples of situations when your balance becomes worse?
- Can you give examples on when your balances improves?
- How does balance affect your ability to do things in your everyday life?
- Can you describe any specific household activities or things that you do at home that are affected by your balance?
- Can you describe some special activities outdoors that you think are harder to do due to your balance?
- Have you experienced that your balance has made it harder to participate in any social activities?
- Are there things in your everyday life do you avoid or have stopped doing due to your balance? If so, why do you think you avoid these things?
- When one gets older it’s more common to fall, what do you think about the risk of falling, is it something that bothers you?
- Another thing that can be troublesome is when one does two things at the same time, how do you experience that? If so, in which situations have you noticed it?
- Is there something that you don’t think that we have spoken about regarding these subjects or that you would like to ask about before we finish?
